# Harnessing Deep Learning to Analyze Cryptic Morphological Variability of *Marchantia polymorpha*

**DOI:** 10.1093/pcp/pcad117

**Published:** 2023-10-05

**Authors:** Yoko Tomizawa, Naoki Minamino, Eita Shimokawa, Shogo Kawamura, Aino Komatsu, Takuma Hiwatashi, Ryuichi Nishihama, Takashi Ueda, Takayuki Kohchi, Yohei Kondo

**Affiliations:** Quantitative Biology Research Group, Exploratory Research Center on Life and Living Systems (ExCELLS), National Institutes of Natural Sciences, 5-1 Higashiyama, Myodaiji-cho, Okazak, Aichii, 444-8787 Japan; Division of Cellular Dynamics, National Institute for Basic Biology, Nishigonaka 38, Myodaiji, Okazaki, Aichi, 444-8585 Japan; Graduate School of Biostudies, Kyoto University, Kitashirakawa-Oiwakecho, Sakyo, Kyoto, 606-8502 Japan; Graduate School of Biostudies, Kyoto University, Kitashirakawa-Oiwakecho, Sakyo, Kyoto, 606-8502 Japan; Graduate School of Biostudies, Kyoto University, Kitashirakawa-Oiwakecho, Sakyo, Kyoto, 606-8502 Japan; Division of Cellular Dynamics, National Institute for Basic Biology, Nishigonaka 38, Myodaiji, Okazaki, Aichi, 444-8585 Japan; Graduate School of Biostudies, Kyoto University, Kitashirakawa-Oiwakecho, Sakyo, Kyoto, 606-8502 Japan; Department of Applied Biological Science, Faculty of Science and Technology, Tokyo University of Science, 2641 Yamazaki, Noda, Chiba, 278-8510 Japan; Division of Cellular Dynamics, National Institute for Basic Biology, Nishigonaka 38, Myodaiji, Okazaki, Aichi, 444-8585 Japan; Department of Basic Biology, SOKENDAI (The Graduate University for Advanced Studies), Nishigonaka 38, Myodaiji, Okazaki, Aichi, 444-8585 Japan; Graduate School of Biostudies, Kyoto University, Kitashirakawa-Oiwakecho, Sakyo, Kyoto, 606-8502 Japan; Quantitative Biology Research Group, Exploratory Research Center on Life and Living Systems (ExCELLS), National Institutes of Natural Sciences, 5-1 Higashiyama, Myodaiji-cho, Okazak, Aichii, 444-8787 Japan; Division of Quantitative Biology, National Institute for Basic Biology, National Institutes of Natural Sciences, 5-1 Higashiyama, Myodaiji-cho, Okazaki, Aichi, 444-8787 Japan; Department of Basic Biology, School of Life Science, SOKENDAI (The Graduate University for Advanced Studies), 5-1 Higashiyama, Myodaiji-cho, Okazaki, Aichi, 444-8787 Japan

**Keywords:** Artificial intelligence, Image analysis, *Marchantia polymorpha*, Sexual dimorphism, Visual explanation

## Abstract

Characterizing phenotypes is a fundamental aspect of biological sciences, although it can be challenging due to various factors. For instance, the liverwort *Marchantia polymorpha* is a model system for plant biology and exhibits morphological variability, making it difficult to identify and quantify distinct phenotypic features using objective measures. To address this issue, we utilized a deep-learning-based image classifier that can handle plant images directly without manual extraction of phenotypic features and analyzed pictures of *M. polymorpha*. This dioicous plant species exhibits morphological differences between male and female wild accessions at an early stage of gemmaling growth, although it remains elusive whether the differences are attributable to sex chromosomes. To isolate the effects of sex chromosomes from autosomal polymorphisms, we established a male and female set of recombinant inbred lines (RILs) from a set of male and female wild accessions. We then trained deep learning models to classify the sexes of the RILs and the wild accessions. Our results showed that the trained classifiers accurately classified male and female gemmalings of wild accessions in the first week of growth, confirming the intuition of researchers in a reproducible and objective manner. In contrast, the RILs were less distinguishable, indicating that the differences between the parental wild accessions arose from autosomal variations. Furthermore, we validated our trained models by an ‘eXplainable AI’ technique that highlights image regions relevant to the classification. Our findings demonstrate that the classifier-based approach provides a powerful tool for analyzing plant species that lack standardized phenotyping metrics.

## Introduction

Plant phenotypes are influenced by genetic and environmental factors, resulting in differences in shape, color and generation of specific organs. Although the characterization of such phenotypes is a fundamental aspect of plant biology, it often poses considerable challenges. For instance, the liverwort *Marchantia polymorpha*, an emerging model system in plant biology (Kohchi et al. 2021, Bowman et al. 2022), has phenotypic traits that are not easily quantifiable. *Marchantia polymorpha* grows as a flattened creeping thallus that periodically bifurcates from the apical notch (Shimamura 2016, Solly et al. 2017). The continuous architecture is variable and thus hinders the identification and quantification of distinct phenotypic features using objective measures. This difficulty makes potentially significant phenotypes cryptic, which means they are recognizable to the human eye but not available for quantitative and statistical analysis. Therefore, developing a method to handle such cryptic phenotype features would facilitate the understanding of *M. polymorpha* and other plant species that differ from well-characterized model organisms.

Phenotypic characterization and comparison using handpicked features, such as the aspect ratio of leaves, is a classic yet effective method. However, such features often lack the necessary expressiveness to describe diverse biological forms. This has led to the development of sophisticated shape and color analysis techniques, often utilizing computer-assisted image analysis (Chitwood and Sinha 2016). For example, Fourier-based analysis allows the decomposition of an arbitrary contour shape into a sum of periodic components with different frequencies and has been applied to characterize cell and tissue shapes. Additionally, the characterization of surface properties such as color and texture has been an active research field to understand, for example, floral evolution driven by the color preferences of insects (Ohashi et al. 2015). However, it remains challenging to analyze shape, color and texture information in a unified manner. Consequently, the discriminative ability of phenotyping methods for image datasets is often inferior to that of the human eye. A research direction for resolving this issue would be to employ neural-network-based image classifiers, which have produced promising results (Singh et al. 2018). Furthermore, Akagi et al. adopted not only deep-learning-based classifiers but also ‘eXplainable AI (XAI)’ techniques to visualize the reasons behind the diagnosis of calyx-end cracking in persimmon fruits (Akagi et al. 2020). This demonstrates the potential of such techniques to augment the cognitive capacity of researchers.

The image-classifier-based approach offers an advantage by eliminating the need for defining specific features. Deep learning models can learn complex features specialized for classification tasks, and thus they are capable of detecting cryptic morphological variability that may not be easily detectable using traditional morphological or visual assessments. However, it should also be noted that there is no guarantee that the deep learning models will use biologically meaningful features in images (Khorram et al. 2021). For example, differences in photographic condition and photographer can lead to subtle changes in the color and brightness of images. If a classifier utilizes such irrelevant image features, it would not have any biological significance, regardless of the accuracy of the classification.

To maximize the above benefit and reduce the risk of using deep learning models, we devised an analysis protocol that includes a validation step of the trained models by human-interpretable feature ablations and XAI techniques to distinguish biologically meaningful classifiers from meaningless ones. Here, we demonstrated the effectiveness of our approach by resolving an open question regarding the morphological variability of *M. polymorpha*. This dioicous plant species often exhibits morphological differences between male and female plants even in accessions collected from the same site, such as Takaragaike accessions (male Tak-1 and female Tak-2) (Bowman et al. 2017) and Australian/Melbourne (Aus) accessions (Flores-Sandoval et al. 2015). However, it remains elusive whether the differences in these wild accessions arise from sex chromosomes. In this study, to isolate the effects of sex chromosomes from autosomal polymorphisms, we established male and female recombinant inbred lines (RILs) from Tak-1 and Tak-2. We then utilized deep learning models to classify the sexes from pictures of the RILs and the wild accessions in gemmalings during the first week of development. To achieve this, we adopted ResNet50 (He et al. 2016), a model architecture that has been proven to be highly effective in image classification. Furthermore, we validated the trained models by an XAI technique that highlights image regions relevant to the classification. Our study paves the way for the application of deep learning models for analyzing plant species that lack standardized phenotyping metrics.

## Results

### Establishment of RILs Rit-1/Rit-2

Common laboratory accessions, such as Tak-1/Tak-2 and Aus male and female lines, were collected as independent plants and therefore possess polymorphisms between the counterparts.

**Fig. 1:**
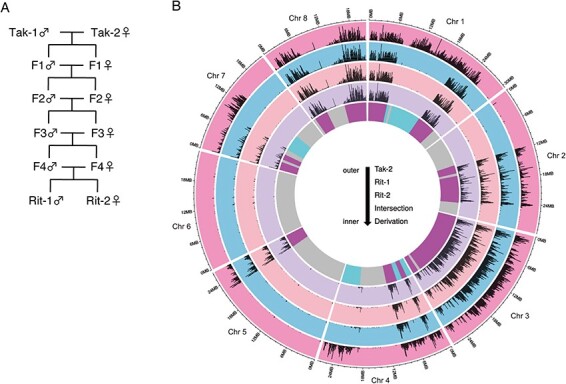
Establishment of RILs. (A) Inbreeding scheme to generate Rit-1 (male) and Rit-2 (female). (B) A Circos plot showing the derivation of genomic regions in RILs’ autosomes. Outer tracks (tracks 1–4) show polymorphism frequencies compared to Tak-1: track 1, Tak-2; track 2, Rit-1; track 3, Rit-2; and track 4, intersection of Tak-2, Rit-1 and Rit-2. The most inner track depicts the predicted derivation of genomic regions in Rit-1/Rit-2. Cyan, regions derived from Tak-1; magenta, those from Tak-2; gray, those shared between Tak-1 and Tak-2.

To reduce autosomal polymorphisms, we generated RILs. Briefly, we first crossed male Tak-1 and female Tak-2 to obtain the F1 generation. We then inbred the siblings four times to obtain F5 generation siblings, which were named ‘Recombinant inbred line derived from Takaragaike (Rit)’, Rit-1 (male) and Rit-2 (female) (**Fig. 1A**). We sequenced the whole genome of Rit-1 and Rit-2 and mapped them, together with Tak-2, to the genome of Tak-1 to call variants (**Fig. 1B**). Rit-1 and Rit-2 shared the same polymorphism patterns in the autosomes. Of the autosomal regions, 57.0% did not contain a significant number of polymorphisms between Tak-1 and Tak-2 (**Supplementary Table S1**), which may have resulted from a natural cross(es) before the collection of Tak-1 and Tak-2. Of the autosomal regions, 30.7% contained a significant number of polymorphisms between Tak-1 and Tak-2 as well as that between Tak-1 and Rit-1 or Rit-2 (**Supplementary Table S1**), indicating that this region was derived from Tak-2 and the rest (12.3%) from Tak-1. These data indicate the establishment of Tak-1/Tak-2-derived RILs.

### Deep learning for sex classification of *M. polymorpha* based on gemmaling images

**Fig. 2:**
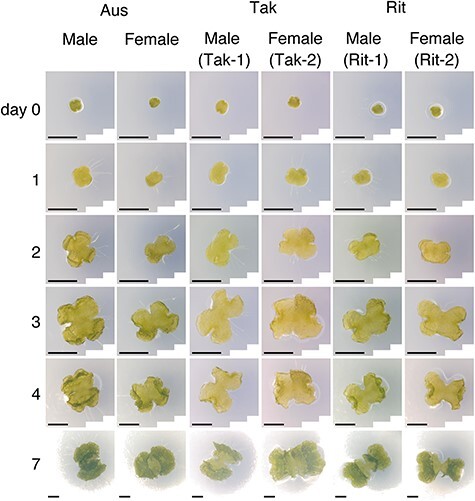
Representative images of early gemmalings from Aus, Tak-1/Tak-2 and Rit-1/Rit-2. Rows: developmental day of gemmalings. Columns: accession and sex. Scale bars, 1 mm.

To acquire images of *M. polymorpha* gemmalings, we used male Rit-1 and female Rit-2, their parental male Tak-1 and female Tak-2, and male/female of another wild accession Aus. We included Aus accession to provide another example exhibiting morphological differences between male and female plants collected at the same site. **Fig. 2** shows representative images of the lines and developmental days. The number of male/female images was approximately 100/100 for the Tak-1/Tak-2 and Aus, and approximately 300/300 for Rit-1/Rit-2 (see **Supplementary Table S2** for details). Images were acquired on days 0, 1, 2, 3, 4 and 7 after planting gemmae. These images were processed and used for further analysis.

**Fig. 3:**
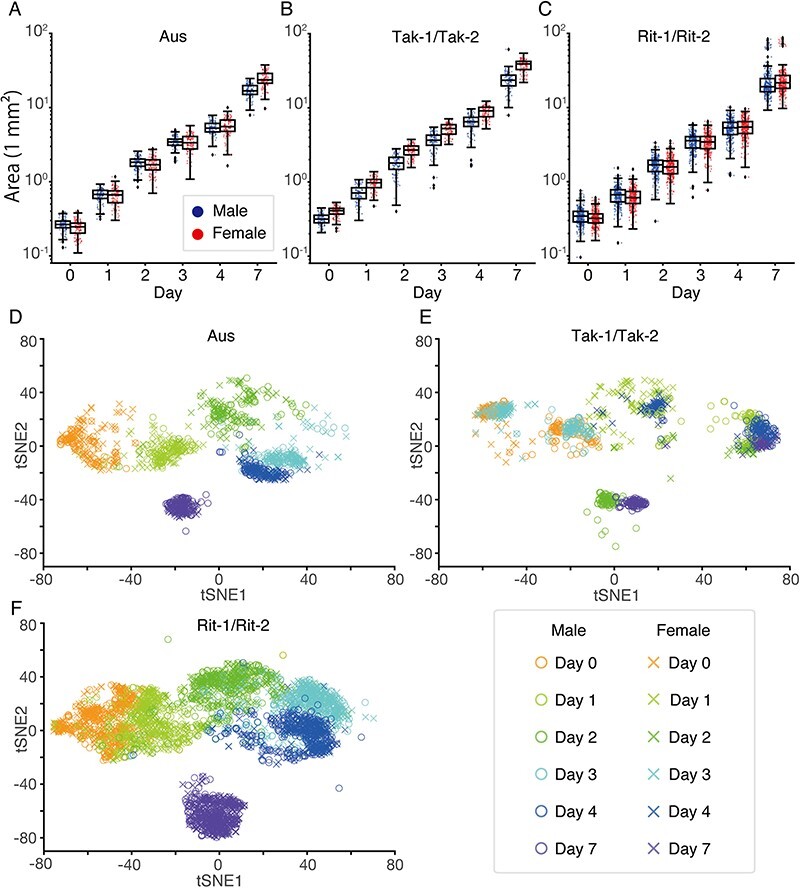
Basic analysis of gemmaling image dataset. (A–C) Quantification of the area of aerial part of gemmalings of Aus (A), Tak-1/Tak-2 (B) and Rit-1/Rit-2 (C). The horizontal axis represents the day after planting gemmae. A box plot is overlaid with individual data points. Colored dots represent individual images. The box plot displays the median (line within the box), the lower and upper quartiles (box) and largest and smallest data points within the interval of 1.5 times the interquartile range from the box (whiskers). Black dots represent outliers. (D–F) Image clustering using t-SNE for Aus (D), Tak-1/Tak-2 (E) and Rit-1/Rit-2 (F). Each point represents a different image.

First, we quantified the simplest morphological feature, the area of the gemmalings (**Fig. 3A****–****C**). In Tak-1/Tak-2, Tak-2 (female) was larger than Tak-1 (male) from day 0. Aus males and females were similar in size up to day 4, but females became larger at day 7. Rit-1/Rit-2 did not differ in size throughout the examined period. We quantitatively validated the findings through the calculation of the unbiased Cohen’s *d*, an estimate for the mean difference normalized by the standard deviation (SD; Gurevitch and Hedges 1993). The calculated values, summarized in **Supplementary Table S3**, evaluate male-to-female differences in areas relative to individual variations.

Second, to visualize the dataset, we conducted dimension reduction using image feature extraction and *t*-distributed stochastic neighbor embedding (t-SNE) (Maaten and Hinton 2008). The image feature extraction was achieved by a pretrained deep neural network, ResNet50 (He et al. 2016). For individual images, we extracted 2,048-dimensional features from the last average pooling layer of the ResNet50. Then, we applied t-SNE to the feature vectors of all developmental days and accessions. **Fig. 3D****–****F** displays the clustering of all images, encompassing three lines and six developmental days. The entire dataset was clustered, but for better visualization, the results are presented in separate figures. In Aus (**Fig. 3D**) and Rit-1/Rit-2 (**Fig. 3F**), the images were grouped into clusters according to the day of development; however, those of different sexes were not distinctly placed into separate clusters. In contrast, Tak-1/Tak-2 (**Fig. 3E**) were separated by sex, and the images were periodically embedded according to developmental days: the first cycle (days 0, 1 and 2) and the second cycle (days 3, 4 and 7). These clustering results suggest that the morphological differences between sexes are difficult to discriminate in Aus and Rit-1/Rit-2 but are clearer in Tak-1/Tak-2.

**Fig. 4:**
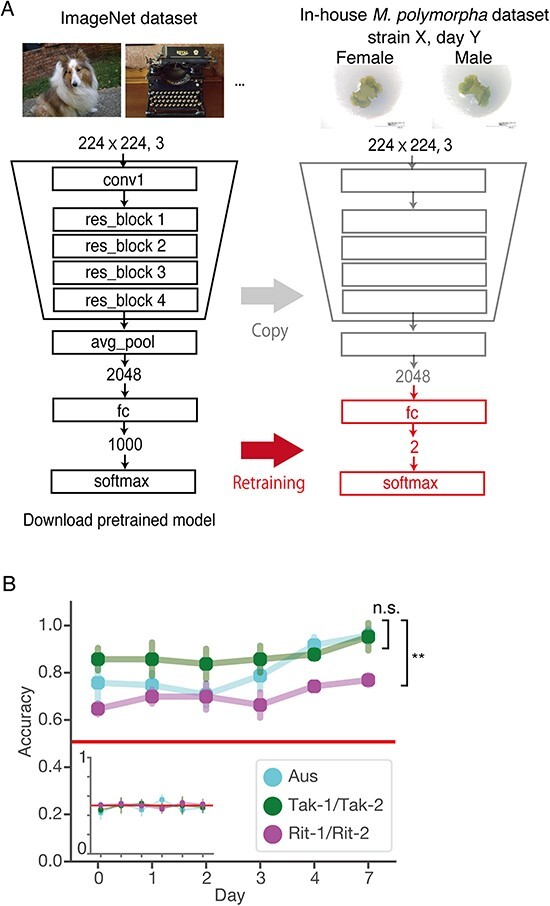
Sex classification of *M. polymorpha* using the ResNet50 architecture. (A) Training scheme of the pretrained ResNet50 model. Only the last fully connected layer was modified and retrained for the sex classification of gemmaling images. Abbreviations: conv, convolutional layer; res_block, residual block; avg_pool, global average pooling; fc, fully connected layer. (B) Test accuracy of sex classification was plotted against developmental days of gemmalings. Points and bars indicate the mean and SD for five independent trials with random training, validation or test splitting for each day. Statistical analyses were performed on day 7. Asterisks indicate a significant difference in a two-tailed Welch’s *t*-test with Bonferroni adjustment, *P* < 0.01; n.s., not significant; *n* = 5. The inset shows negative controls where the sex labels of the images were randomly permuted.

Finally, we trained ResNet50, a well-tested deep learning model for image classification. The model was fed with input information, i.e. images of *M. polymorpha* gemmalings and sex labels associated with each image, to predict the sex of the new images with hidden sex labels. Although we took hundreds of images of gemmalings, the dataset size was much smaller than that of common training datasets, which typically contain millions of images. Because deep learning models have numerous parameters (e.g. 25.6 million in ResNet50), training a model with a small dataset may lead to overfitting, a situation in which a model simply memorizes all training images and corresponding sex labels instead of extracting meaningful morphological traits. To avoid overfitting, we employed a common approach for transfer learning. As illustrated in **Fig. 4A**, we utilized a pretrained ResNet50 model with the ImageNet dataset (Russakovsky et al. 2015) as a starting point and then retrained only the last layer of the model to classify the sex of the plant (see Materials and Methods for further details about the training process) (Donahue et al. 2014, Sharif Razavian et al. 2014).

**Fig. 4B** shows the classification accuracy for the test images, i.e. the accuracy for images not used in the training phase. Test accuracy, unlike training accuracy, helps to detect invalid models that simply memorize training images without learning meaningful features. For each developmental day and set of male and female accessions, the images were independently analyzed using different models; hence, each model addressed the binary classification of male/female. For each classification task, we conducted five independent model training sessions using randomly selected training, validation and test images from the dataset. We used 64% of the images for training, 16% for validation and 20% for testing. As a negative control, we trained the same model based on randomized sex labels (**Fig. 4B**, inset). This confirmed that the test accuracies in the negative control setting were indistinguishable from chance. Detailed evaluation of the trained models is in **Supplementary Figs. S1–S3**.

As shown in **Fig. 4B**, the gemmaling images of Tak-1/Tak-2, compared to those of Aus and Rit-1/Rit-2, were most easily classified, with an accuracy of >90% on day 7. This is consistent with the area quantification (**Fig. 3A****–****C**) and t-SNE clustering (**Fig. 3D****–****F**), which suggests that Tak-1 and Tak-2 are more distinguishable than the other accessions. The classification accuracy for Aus was relatively low on day 0, whereas the accuracy increased substantially after day 2, becoming comparable to that for Tak-1/Tak-2 after day 4. Meanwhile, the trained models encountered difficulty in accurately classifying the test images of Rit-1/Rit-2 and demonstrated the lowest accuracies (from 64.0 ± 2.3% on day 0 to 76.2 ± 2.1% on day 7) among all examined accessions, despite having a larger number of training images compared to the wild accessions. This finding objectively confirms the intuition of researchers that morphological differences between sexes in wild accessions are more distinct and these differences are diminished in the inbred lines.

### Dissecting image regions relevant to the decision-making of trained deep learning models

Deep learning models are capable of utilizing any type of visual property, such as texture, color, shape and the presence of particular objects. However, a model may undesirably recognize irrelevant features such as background medium color, rather than sex-associated morphological differences. To investigate the features that the trained model utilized for classification, we removed certain features from the original images and newly trained the ResNet50 model with the feature-ablated images.

**Fig. 5:**
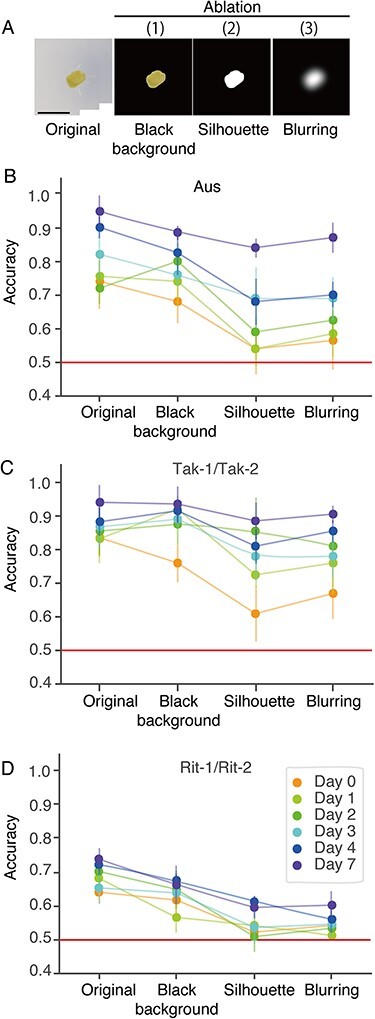
Validation of the trained classifier with feature ablation. (A) Examples of feature-ablated images. (B–D) Plots showing test accuracy against feature-ablation types for Aus (B), Tak-1/Tak-2 (C) and Rit-1/Rit-2 (D). The same ablation procedure was applied to training, validation and test datasets in each model training session. The data points are grouped by developmental days, indicated by the colors. Points and bars represent the mean and SD, respectively, for five independent trials with random training, validation or test splitting.

We conducted three types of feature ablations: (I) masking the background with black, (II) binarization into white foreground and black background and (III) binarization and severe blurring (**Fig. 5A**). For ablation (I), we eliminated information from the background by filling all the background areas in black. If the model recognized features from the background, then the test accuracies would decrease compared to those from the original setup. Thus, the decrease in accuracy quantifies the information from the background. For ablation (II), we binarized images to erase the information on the color and texture of the plant aerial part as well as the information from the background. For ablation (III), we severely blurred the binarized images to render detailed contours unrecognizable.

**Fig. 5B** **–** **D** shows the test accuracies of the feature-ablation experiments for Aus (**Fig. 5B**), Tak-1/Tak-2 (**Fig. 5C**) and Rit-1/Rit-2 (**Fig. 5D**). When comparing the accessions, the classification accuracies for Rit-1/Rit-2 were lower than those for the wild accessions and dropped to near-chance rates under ablations (II) and (III). The results confirmed that Rit-1 and Rit-2, compared with the wild accessions, show smaller differences between male and female plants. Previous studies have shown that deep learning models tend to use not only foreground regions but also background for image classification (Geirhos et al. 2020, Moayeri et al. 2022). We also found modest decreases in the test accuracy under most conditions after ablation (I) in Aus and Rit-1/Rit-2, indicating that the trained models partially utilized background information irrelevant to plant morphology. The test accuracies for Tak-1/Tak-2, except day 0, were little affected by ablation (I), possibly because noticeable differences between gemmalings of Tak-1 and Tak-2 obviate the need to rely on background information. In all accessions, the test accuracies remained well above the chance rate after ablation (I), and this indicates that the models utilized the information of plant area. Ablation (II), which involved binarization, resulted in degradation of the accuracy in all conditions. This indicates that the color and/or texture of gemmalings are at least equally informative as compared to the background. On the other hand, ablation (III) did not lead to accuracy reduction. Previous feature-ablation experiments have shown that ImageNet-pretrained models are capable of classifying silhouette images if there are shape differences (Kubilius et al. 2016, Geirhos et al. 2019). Thus, the comparable accuracies under ablation (II) and ablation (III) indicate that the contour shape of gemmalings did not contribute to the classification. However, this result does not completely eliminate the possibility that the correlation between contour shape and color/texture might be informative for the classification since the correlation was already lost after ablation (II). Note that the gemmalings of Aus and Tak-1/Tak-2 exhibited the area differences between the sexes (**Fig. 3A, B**), thus it is not surprising that the images of wild accessions were still classifiable after severe blurring, ablation (III).

The above analysis raised a concern that the background ablation possibly influences the results in **Fig. 4B**. Thus, we plotted the test accuracies for the images after ablation (I) against the developmental days as in **Fig. 4B** and confirmed that the conclusion remained unaffected (**Supplementary Fig. S4**).

### XAI methods for model validation

For further validation, it would be useful to analyze the parameters of the trained models. However, such an analysis is intractable because of the sheer complexity of deep learning models. To make these models transparent and interpretable, post hoc model-analysis methods known as ‘XAI’ have been developed in the past decade. To visualize the relevant regions for the decision-making of the trained models, we employed Grad-CAM (Selvaraju et al. 2017), which is one of the most well-tested XAI methods for image classifier models. Grad-CAM generates a ‘class-discriminative localization map’, which is a heatmap superimposed on an input image that highlights discriminative regions containing critically important features for classification.

Grad-CAM heatmaps visualized whether discriminative features were located in the gemmalings themselves or in the background. If the models appropriately pay attention to plant region, discriminative features should appear in the gemmaling area. In contrast, the Grad-CAM heatmaps on the background instead of the gemmalings indicate that the models utilized irrelevant features.

**Fig. 6:**
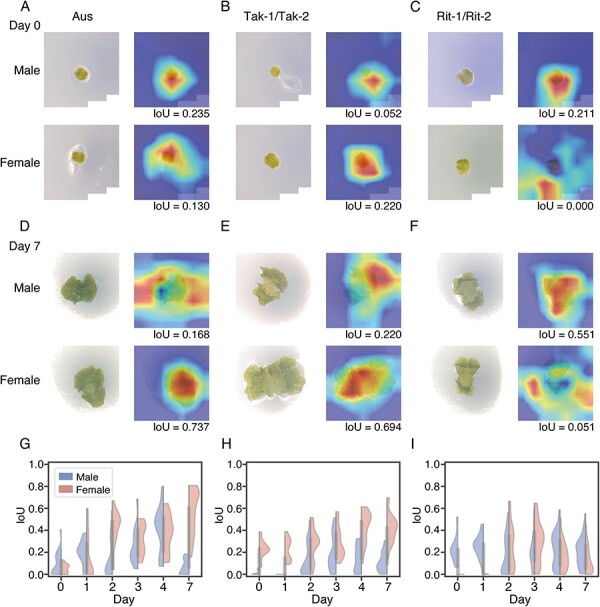
Grad-CAM heatmaps visualize image regions relevant to classification. (A–F) Representative Grad-CAM heatmaps for Aus day 0 (A), Tak-1/Tak-2 day 0 (B), Rit-1/Rit-2 day 0 (C), Aus day 7 (D), Tak-1/Tak-2 day 7 (E) and Rit-1/Rit-2 day 7 (F). IoU scores quantify the degree of overlap between the Grad-CAM heatmaps and the genmmalings. (G–I) Distribution of quantified overlap between the Grad-CAM heatmap and the gemmaling with the IoU score. Violin plots show IoU scores on correctly predicted images of (G) Aus, (H) Tak-1/Tak-2 and (I) Rit-1/Rit-2. Points and bars indicate the median and interquartile range, respectively. The left and right sides of each violin plot represent male and female, respectively.

**Fig. 6A** **–** **F** shows representative Grad-CAM heatmaps for predicted sex for test images of all accessions on days 0 and 7. Here, we focused on correctly classified images. To avoid displaying cherry-picked results upon selecting a single representative heatmap among the 20 (Aus and Tak-1/Tak-2) and 60 (Rit-1/Rit-2) test images for each developmental day and each sex, we quantified the degree of confidence that the models had in each classification and used the images with the highest confidence scores. We found that the representative heatmaps often appeared on the aerial part of the gemmaling, while other cases imply problematic attention to, for example, a scratch on the culture medium (**Fig. 6B**, top right).

Next, we calculated Intersection over Union (IoU), also known as the Jaccard coefficient, to summarize the image-wise explanations across the test images. The IoU score is a metric for measuring the degree of overlap between two regions of interest, where 1 (maximum) indicates a pixel-perfect match and 0 (minimum) indicates no overlapping area. **Fig. 6G****–****I** shows the IoU scores between the Grad-CAM heatmaps and the aerial parts of the gemmalings for all test images (see **Supplementary Fig. S5** for the heatmap area normalized by the whole image area). Until day 1, the IoU scores on males and/or females were close to zero, suggesting that the models responded to nuisance features such as background medium color. Meanwhile, from days 3 to 7, the Grad-CAM heatmaps overlapped with the gemmalings in both male and female images. The exception to this trend is on day 7 of Aus male when the heatmaps appeared mostly on the culture medium. Interestingly, all accessions exhibited a transition in the IoU distribution around day 2, although the time courses of classification accuracy (**Fig. 4B**) were largely different among the accessions.

**Fig. 7:**
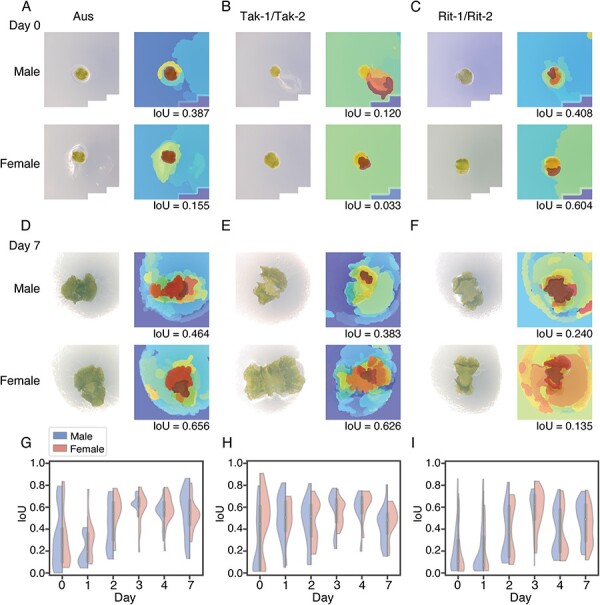
XRAI heatmaps visualize image regions relevant to classification. (A–F) Representative XRAI heatmaps for Aus day 0 (A), Tak-1/Tak-2 day 0 (B), Rit-1/Rit-2 day 0 (C), Aus day 7 (D), Tak-1/Tak-2 day 7 (E) and Rit-1/Rit-2 day 7 (F). IoU scores quantify the degree of overlap between the Grad-CAM heatmaps and the genmmalings. (G–I) Distribution of quantified overlap between the XRAI heatmap and the gemmaling with the IoU score. Violin plots show IoU scores on correctly predicted images of (G) Aus, (H) Tak-1/Tak-2 and (I) Rit-1/Rit-2. Points and bars indicate the median and interquartile range, respectively. The left and right sides of each violin plot represent male and female, respectively.

Although Grad-CAM provides valuable insights with regard to relevant regions for image classification, the heatmaps are often too coarse for dissecting subregions within an organism. Besides, such coarse heatmaps can make IoU scores misleading. Thus, higher-resolution visual explanation methods, such as Layer-wise Relevance Propagation (Bach et al. 2015) and a region-based attribution method, XRAI (Kapishnikov et al. 2019), would be useful for further validation. To this end, we conducted the same analysis as in **Fig. 6**, but using XRAI. **Fig. 7A****–****F** shows representative XRAI heatmaps for predicted sex for test images of all accessions on days 0 and 7. As expected, we found that the XRAI heatmaps exhibited more prominent localization to the gemmalings. Note that XRAI also detected the problematic attention to the scratch on the culture medium (**Fig. 7B**, top right), illustrating the consistency between Grad-CAM and XRAI results. Then, we calculated the IoU scores for the test images (**Fig. 7G****–****I**) (see **Supplementary Fig. S6** for the heatmap area normalized by the total image area). The overlap between the XRAI heatmaps and the aerial part of gemmalings increases along with the developmental days. In comparison with the Grad-CAM results, the transition on day 2 was less clear for Tak-1/Tak-2. In addition, the IoU scores of XRAI, unlike those of Grad-CAM, did not exhibit significant differences between males and females. Taken together, we confirmed that the decision-making of the trained models was based on the gemmalings rather than nuisance background features after day 2.

### Transferability of Tak-1/Tak-2 and Rit-1/Rit-2 classifiers

**Fig. 8:**
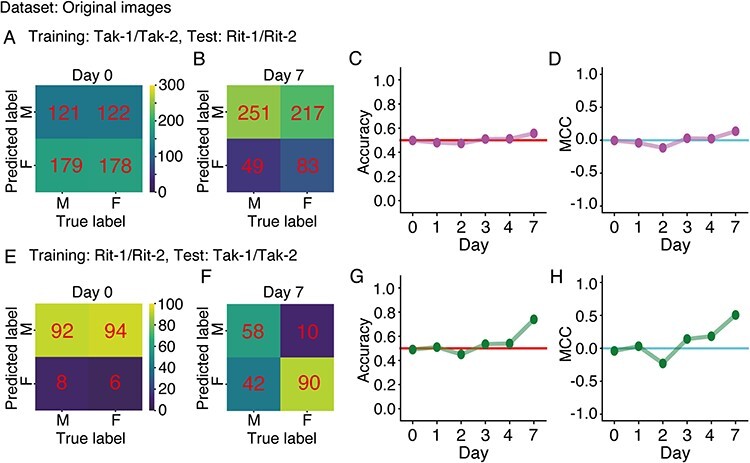
Performance of transfer prediction. Original non-ablated images were used in training, validation and testing. (A–D) Performance of Tak-1/Tak-2 classifiers on Rit-1/Rit-2 images. Confusion matrices for true sex labels of Rit-1/Rit-2 plants (columns) and predicted sex labels (rows) for day 0 (A) and day 7 (B). M and F denote male and female, respectively. Numbers in the confusion matrices indicate the numbers of Rit-1/Rit-2 images in the corresponding categories. If the prediction is perfectly accurate for both males and females, the matrix should be diagonal. Prediction accuracy (C) and MCC (D) were plotted against developmental days of gemmalings. (E–H) Performance of Rit-1/Rit-2 classifiers on Tak-1/Tak-2 images. Confusion matrices for true sex labels of Tak-1/Tak-2 plants (columns) and predicted sex labels (rows) for day 0 (E) and day 7 (F). Prediction accuracy (G) and MCC (H) were plotted against developmental days of gemmalings.

We have focused on the test accuracy of classifiers for the images of the same accessions as in the training data, and thereby we have found that accession-specific morphological differences in Tak-1/Tak-2 were reduced in Rit-1/Rit-2. Additionally, it would also be interesting to examine the accuracy of Tak-1/Tak-2 (Rit-1/Rit-2) classifiers on Rit-1/Rit-2 (Tak-1/Tak-2) images. For example, if the small yet detectable differences in Rit-1/Rit-2 were inherited from Tak-1/Tak-2, the classifiers trained on Rit-1/Rit-2 images would be able to classify Tak-1/Tak-2 images. We conducted such transfer-prediction experiments. **Fig. 8A****–****D** shows the prediction performances of Tak-1/Tak-2 classifiers on Rit-1/Rit-2 images on each developmental day, while **Fig. 8E****–****H** shows those of the Rit-1/Rit-2 classifiers on Tak-1/Tak-2 images (see **Supplementary Fig. S7** for details). We found that, on all developmental days, the accuracies of Tak-1/Tak-2 classifiers on Rit-1/Rit-2 images were poor. On the other hand, the Rit-1/Rit-2 classifier on day 7 (**Fig. 8F**) was able to classify Tak-1/Tak-2 images with an accuracy of 0.74 (**Fig. 8G**) and Matthew’s correlation coefficient (MCC) of 0.507 (**Fig. 8H**). The asymmetric transfer-prediction performances between Tak-1/Tak-2 and Rit-1/Rit-2 classifiers were robustly reproduced under ablation (I) and ablation (II), i.e. background-ablated and silhouette images (**Supplementary Figs. S8**, **S9**). The results suggest that the remaining morphological differences in Rit-1/Rit-2 were inherited from parental Tak-1/Tak-2; in other words, the morphological differences in Tak-1/Tak-2 did not completely disappear in Rit-1/Rit-2. On the other hand, concerning the poor performance of Tak-1/Tak-2 classifiers on Rit-1/Rit-2 images, we hypothesized that the Tak-1/Tak-2 classifiers relied on easy-to-learn differences such as area and ignored the other features, which is a common problem in deep-learning-based image classifiers (Geirhos et al. 2020).

## Discussion

In this study, we proposed a deep-learning-based approach to detect and quantify cryptic morphological variability in *M. polymorpha*. We have demonstrated the effectiveness of our approach by analyzing the differences observed in gemmalings of different sexes during the first week of development. To isolate the contributions of sex chromosomes, we established RILs, Rit-1 and Rit-2, from the wild accessions, Tak-1 and Tak-2. To construct a dataset for deep learning, we acquired images of gemmalings of Rit-1/Rit-2, Tak-1/Tak-1 and male/female of another wild accession, Aus. We observed that deep-learning-based ResNet50 successfully classified male and female gemmalings of the wild accessions with >90% test accuracy, while it could not reliably predict the sex of the RILs (**Fig. 4B**). This implies that the RILs have lost morphological differences present in parental Tak-1 and Tak-2, indicating that they are most likely attributable to autosomal variations rather than solely determined by sex chromosomes. Notably, the parental Tak-1 and Tak-2 had identical sequences in >50% of their genomes (**Fig. 1**), which shows that Tak-1 and Tak-2 had been already inbred at the time of collecting, despite visible morphological differences even within the first week of gemmaling growth. This also highlights the importance of carefully constructed inbred lines; thus, Rit-1 and Rit-2 serve as an appropriate model system for investigating sexual dimorphism. Our approach is particularly important for quantifying the degree of phenotypic loss resulting from genetic changes, because the deep-learning-based classifiers eliminate the need for manual feature definition, thus bypassing the ‘devil’s proof’ that manually defined features may be inadequate for capturing phenotypic changes.

We trained separate classifiers for each developmental day and set of male and female accessions. The model design enabled us to conduct the transfer-prediction experiments (**Fig. 8**), which gave insights into the features learned from Tak-1/Tak-2 and Rit-1/Rit-2 images. An alternative design is to train a unified model on all images possibly with supplemental inputs such as developmental days. Such approaches are data-efficient and simpler to analyze and hence would be useful for further analysis.

To validate whether the models learned to use features of biological importance, we adopted feature-ablation experiments and XAI methods such as Grad-CAM and XRAI (**Figs. 5****–****7**). As a result, we confirmed that after a few days of growth, the sex classifications of the trained models depended on the plant body rather than irrelevant features such as scratches on the medium surface. An interesting research direction would be to identify the exact morphological features responsible for this classification. Another promising research direction is the so-called ‘Human-in-the-Loop’ approach. For example, in the Clustering-Aided Rapid Training Agent framework (Kutsuna et al. 2012), a model optimizes a set of features based on human feedback, simultaneously leading to high accuracy and interpretability.

Our approach based on the classifier has the advantage of being applicable to other modalities, such as fluorescence microscopy and infrared thermography. In particular, recent advancements in fluorescence/luminescence reporters have allowed us to acquire diverse image data for which optimal methods of characterization and quantification have not yet been established. The procedures developed here should facilitate a wide range of applications of deep-learning-based methods in plant biology.

## Materials and Methods

### Establishment of the RILs of *M. polymorpha*

The accessions Tak-1 and Tak-2 (Ishizaki et al. 2008) were used as original male and female strains, respectively, for the generation of RILs. All plants of Tak-1, Tak-2 and their offsprings were cultured on half-strength Gamborg’s B5 (1/2 B5) medium (Gamborg et al. 1968) with 1% agar under continuous light (50∼60 µmol photons m^−2^ s^−1^) at 22°C.

To cross male and female plants, gemmae were aseptically incubated on 1/2 B5 medium for >2 weeks and then incubated in a container, ECO2box (E1654, Duchefa Biochemie B.V., Haarlem, Netherland), containing 1/2 B5 medium under sexual-organ induction conditions, i.e. continuous white light (50∼60 µmol photons m^−2^ s^−1^) supplemented with far-red light (30 µmol photons m^−2^ s^−1^) at 18°C. After archegoniophores and antheridiophores had been formed, sperm were collected from antheridia as suspension in sterile water and put onto archegoniophores three times a week until sporangia were visibly recognized. Matured sporangia were collected and dried for 1 week at room temperature. Dried sporangia were mixed into sterilized water, then spread onto 1/2 B5 medium plate and grown to the optimal size that is enough for genotyping of sex diagnosis as described previously (Iwasaki et al. 2021).

The spores obtained by the cross between Tak-1 and Tak-2 were designated as the F1 generation. More than 10 male and female F1 individuals were selected. Crosses between more than five sibling couples were performed, and one normal-looking sporangium was collected from each cross. These sporangia were separately spread onto 1/2 B5 medium, and one with enough number of spores and a high germination rate was selected as the F2 generation. These procedures were repeated for several generations. A pair of F5 siblings were defined as the male and female RILs Rit-1 and Rit-2.

### Genome sequencing

Rit-1 and Rit-2 genomic DNAs were purified, and DNA libraries for sequencing were constructed using the NEBNext Ultra II FS DNA Library Prep Kit for Illumina and used to obtain paired-end reads using the Illumina NextSeq500 platform.

### Validation of RILs by polymorphism detection

Fastq files available from SRA-run ID DRR120994 were used for the analysis of Tak-2 polymorphisms. The fastq files of Tak-2, Rit-1 and Rit-2 were trimmed by fastp (v0.12.4) (Chen et al. 2018) and then mapped against Tak-1 genome v6.1 [downloaded from MarpolBase (marchantia.info)] by bwa-mem2 (v2.2.1) (Vasimuddin et al. 2019) at default parameters. Output sam files were sorted by samtools (v1.11) (Li et al. 2009) sort, followed by samtools fixmate with -m option. Samtools sort with -n option was used to sort these bam files before samtools markdup to produce dedupped bam files for following variant calling. Due to the lack of a golden standard list of single-nucleotide polymorphisms (SNPs) in *M. polymorpha* unlike human, we used variants called by GATK (v4.1.3.0) (McKenna et al. 2010) to recalibrate bam files for the second variant call. The first variant call was done by gatk HaplotypeCaller with -pairHMM LOGLESS_CACHING -ERC GVCF -ploidy 1 options followed by the run of GenotypeGVCF with -ploidy 1 option. Hard filters were applied for each SNP and insertion and deletion (INDEL) to filter out low-quality variants by gatk VariantFilteration and SelectVariants—exclude-filtered. For SNP filtering, QD < 2.0, QUAL < 30.0, SOR > 3.0, FS > 60.0, MQ < 40.0, MQRankSum < −12.5 and ReadProRankSum < −8.0 criteria were used. QD < 2.0, QUAL < 30.0 and FS > 20.0 filters were used for INDEL filtering. Output vcf files for SNPs and INDELs were fed to gatk BaseRecalibrator to generate a recalibration table with which gatk ApplyBQSR recalibrated bam files for the second variant call. The second variant call was done as described above to gain vcf files for SNPs and INDELs, respectively. Detected variants were also filtered out by the threshold described in the first step of variant filtration. To produce a Circos plot, we used bcftools (v1.9) (Danecek et al. 2021) norm to normalize the called variants and bcftools isec to detect intersections among Tak-2, Rit-1 and Rit-2. A homemade R script (https://github.com/PMB-KU/Rit-dev) was used to count polymorphisms, including SNPs and INDELs, in every 100 or 1,000 kb window. The regions that contained >100 polymorphisms per 100 kb or 1,000 polymorphisms per 1,000 kb were defined as those with a significant number of polymorphisms in **Supplementary Table S1** or **Fig. 1B**, respectively. The Circos plot was visualized by the shinyCircos package (Yu et al. 2018).

The fasta files of the Rit-1 and Rit-2 genome sequences were created based on SNPs and INDELs using the gatk FastaAlternateReferenceMaker. Filtered reads were mapped against the created genome sequences using bwa-mem2 with default parameters. Coverage was calculated using the bedtools (v2.30.0) (Quinlan and Hall 2010) genomecov, and regions with 0 coverage were masked with N using the bedtools maskfasta. These masked fasta files were registered as PRJDB15748 in the International Nucleotide Sequence Database Collaboration.

### Image acquisition

Tak-1/Tak-2, Rit-1/Rit-2 and male and female Aus accessions were cultured from gemmae on 1/2 B5 medium containing 1.0% agar at 22°C under continuous white light. Images were acquired by the digital microscope KH-7700 (Hirox, Tokyo, Japan) equipped with the lens MXG-2016Z (days 0∼3: ×60, day 4: ×40, day 7: ×20). The dataset includes 199–200 plants for Aus and Tak-1/Tak-2 and 600–602 for Rit-1/Rit-2, with the same numbers of female and male individuals, except for 99 females and 100 males in Aus 7-day-old gemmalings (see **Supplementary Table S2** for details). The time points were for days 0, 1, 2, 3, 4 and 7 after planting gemmae.

### Data visualization of gemmaling images

For dimension reduction of the gemmaling images, we first preprocessed the images with ImageNet-pretrained ResNet50 (ResNet50_Weights.IMAGENET1K_V2 from the torchvision library) as a feature extractor. Specifically, we extracted image features from the last global average pooling layer; hence, the original dataset images (1,200 × 1,600 × 3) were reduced to 2,048-dimensional vectors. Then, for t-SNE clustering, we used a Python implementation from the scikit-learn library, ‘sklearn.manifold.TSNE’ (random_state: 1,000,000, perp: 30, n_iter: 1,000).

### Training and validation of deep learning models

We preprocessed images before feeding them to deep learning models using the ‘transform’ function in the torchvision library from the PyTorch project (see **Supplementary Table S4** for the versions of the libraries used in this study). To construct the training dataset, we began by center-cropping the original images with a crop size of 1,200 pixels to remove the excess background regions. Next, we erased the scale bar and augmented the images by flipping and rotating them. Finally, we resized the images to 300 × 300 pixels. Then, the pixel intensity range was transformed from [0, 255] to [0, 1], and the red, green, and blue intensities of each image were normalized by using the mean [0.485, 0.456, 0.406] and SD [0.229, 0.224, 0.225]. For the validation/test dataset, we used the same preprocessing steps except that we did not apply flipping or rotation. **Supplementary Table S5** provides a pseudocode for the data augmentation.

We used ImageNet-pretrained ResNet50 (ResNet50_Weights.IMAGENET1K_V2 from the torchvision library) as the base model. The penultimate layer was customized for binary classification and only the parameters of the layer were trained. The images were randomly split into training, validation and test sets at a ratio of 64:16:20. We adopted the Adam optimizer (learning rate: 0.001, batch size: 32, epochs: 500) and used the parameters that achieved the maximum accuracy on the validation set during training.

We adopted the scikit-image library to implement the image modifications in **Fig. 5**. First, we blurred the images using a Gaussian filter (bandwidth = 10 pixels) and created binary masks using Otsu’s thresholding. Then, we applied dilation and hole-filling operations in order to make the masks cover the entire regions of gemmalings. Masks were used to eliminate the background/foreground information in the images. A Gaussian filter (bandwidth = 60 pixels) was applied for severe blurring.

### Visual explanation based on XAI methods

We adopted the Grad-CAM method to visualize highly relevant regions for the classification of the trained models (Selvaraju et al. 2017). By using a PyTorch implementation (https://github.com/jacobgil/pytorch-grad-cam, version 1.4.8), we computed the Grad-CAM heatmaps based on the gradients with regard to activations in the last convolutional layer. Then, from the heatmaps for correctly predicted test images for each accession/sex/developmental day, we selected a representative heatmap for the image of the highest value of the unnormalized logit, i.e. the raw output of the last fully connected layer in ResNet50. The logit values were used to quantify the degree of representativeness of the input images, following recent studies on out-of-distribution detection in deep learning models (Hendrycks et al. 2022; Vaze et al. 2022).

We also adopted XRAI (Kapishnikov et al. 2019) to obtain fine-grained visualization of relevant regions for classification. We modified the original XRAI implementation in TensorFlow (https://github.com/PAIR-code/saliency, version 0.2.0) to analyze our PyTorch models. The selection criteria for representative heatmaps were the same as those for Grad-CAM.

We used IoU, also known as the Jaccard coefficient, in order to measure the degree of overlap between the aerial part of gemmalings and the Grad-CAM/XRAI heatmaps. To this end, we binarized the normalized Grad-CAM/XRAI heatmaps with a threshold of 0.5. For the gemmalings, we utilized the silhouette images (**Fig. 5**). For each binarized image and the corresponding heatmap, IoU is defined as the area of intersection between the image and the heatmap normalized by the area of union of the image and the heatmap.

## Supplementary Material

pcad117_Supp

## Data Availability

Image data underlying this article are available in the RIKEN SSBD:repository (Systems Science Biological Dynamics repository) with the https://ssbd.riken.jp/repository/290/. Our code used for training neural networks is available at https://github.com/nyunyu122/Marchantia_sex_classifier. Sequence data are available at PRJDB15748. Lines generated during this study will be shared on reasonable request to T.K. (tkohchi@lif.kyoto-u.ac.jp).
