# Supplementary material for: Harnessing Deep Learning to Analyze Cryptic Morphological Variability of Marchantia polymorpha
Source: Plant Cell Physiol. 2023 Oct 5;64(11):1343–55. doi: 10.1093/pcp/pcad117 (PMC10700009; doi:10.1093/pcp/pcad117)
Supplement: pcad117_Supp [file pcad117_supp.zip › suppl_data/pcp-2023-e-00096-File010.pdf]

Supplementary Materials

Harnessing deep learning to analyze cryptic morphological variability of *Marchantia polymorpha*

Short title
deep learning analysis for cryptic morphology

Corresponding author
Yohei Kondo
Quantitative Biology Research Group, Exploratory Research Center on Life and Living Systems (ExCELLS), National Institutes of Natural Sciences, 5-1 Higashiyama, Myodaiji-cho, Okazaki, Aichi, 444-8787, Japan
Department of Basic Biology, School of Life Science, SOKENDAI (The Graduate University for Advanced Studies), 5-1 Higashiyama, Myodaiji-cho, Okazaki, Aichi, 444-8787, Japan
TEL.: +81-564-59-5235
Email: y-kondo@nibb.ac.jp

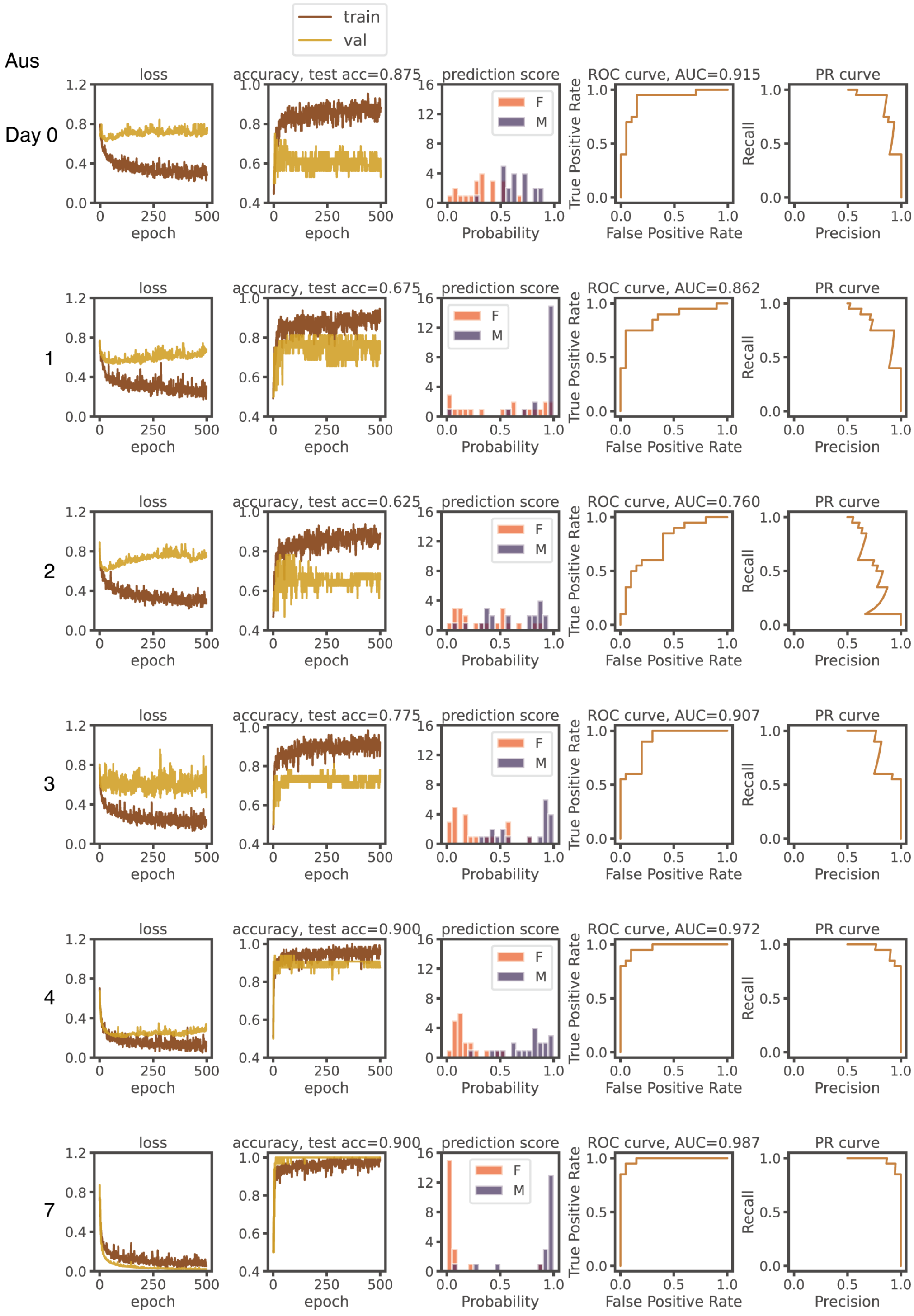

Supplementary Figure S1. Evaluation of the models trained on Aus images. The model with the highest accuracy is shown for each day among models trained on 5 different training/validation/test splits. Rows: developmental days of gemmalings. Columns (left to right): Loss curve, accuracy curve, distribution of prediction scores, ROC curve, and PR curve.

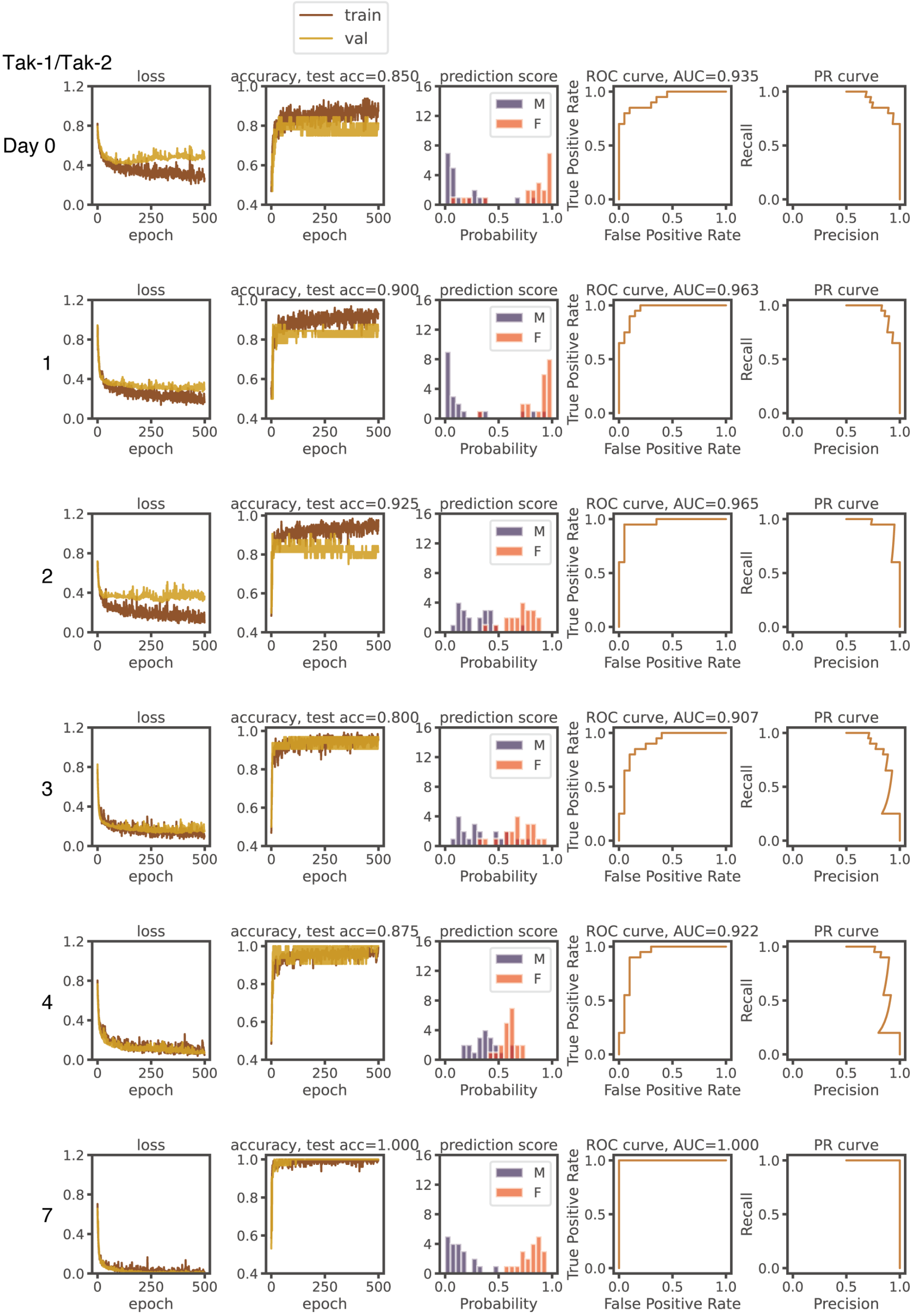

Supplementary Figure S2. Evaluation of the models trained on Tak-1/Tak-2 images. All other settings are the same as in Supplementary Figure S1.

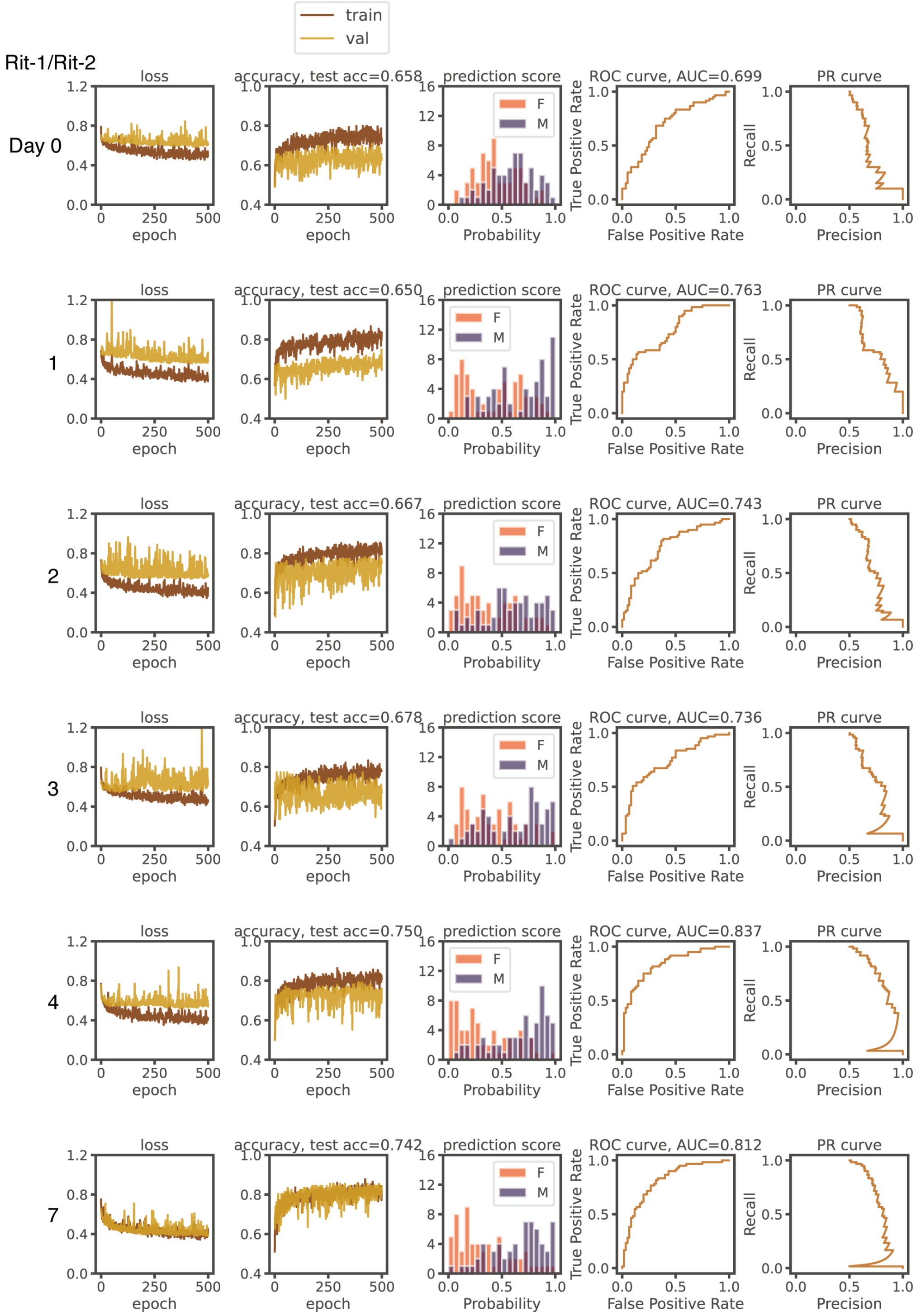

Supplementary Figure S3. Evaluation of the models trained on Rit-1/Rit-2 images. All other settings are the same as in Supplementary Figure S1.

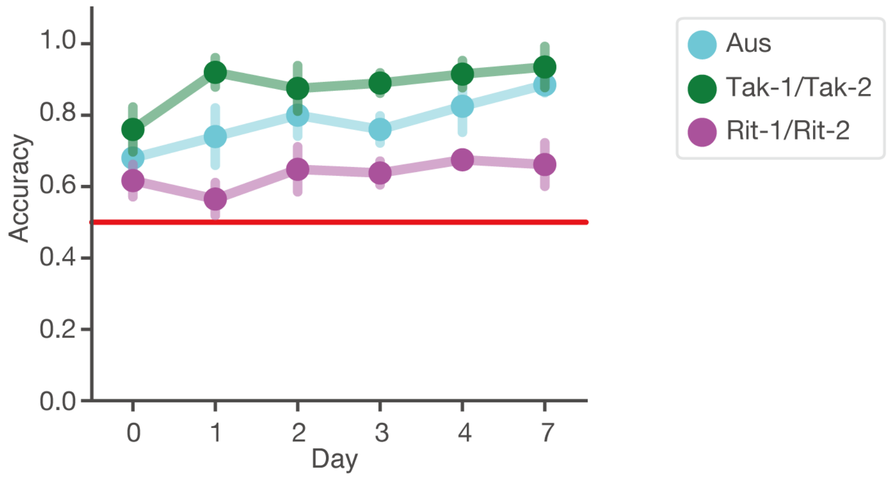

Supplementary Figure S4. Test accuracy for the background-ablated images, i.e., Ablation (1), was plotted against developmental days of gemmalings. Points and bars indicate mean and SD for 5 independent trials with random training/validation/test splitting for each day.

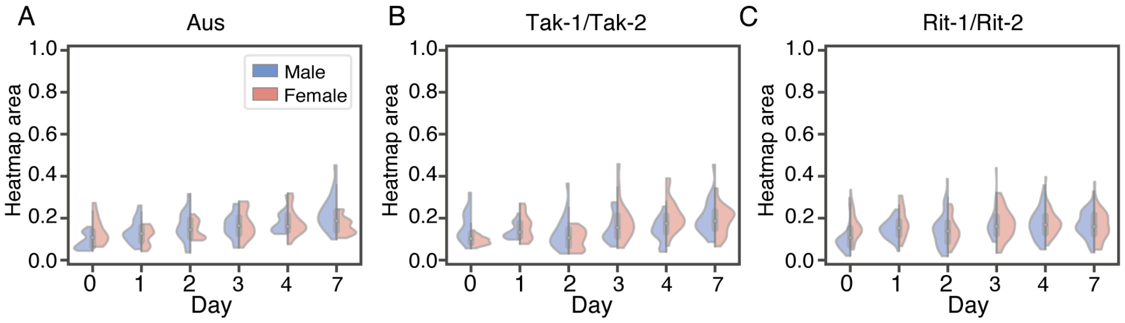

Supplementary Figure S5. Violin plots displaying areas of Grad-CAM heatmaps normalized by the whole-image area for (A) Aus, (B) Tak-1/Tak-2, (C) Rit-1/Rit-2. Scores for correctly-predicted images are shown. Pale blue and pink represent male and female, respectively.

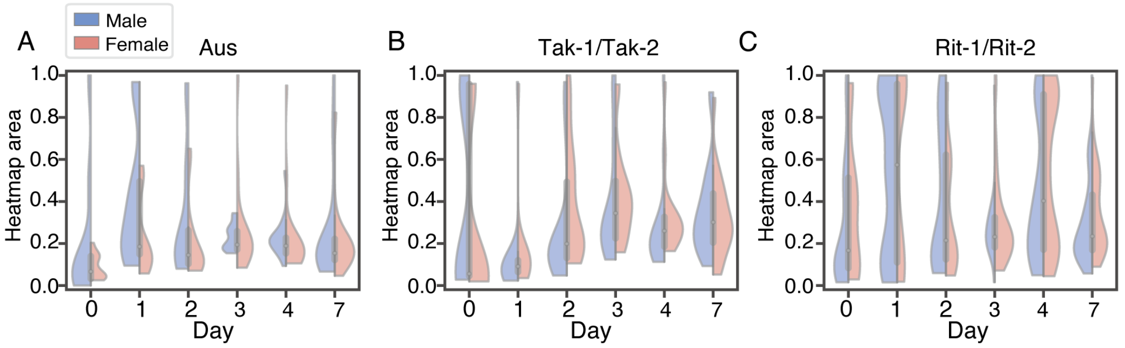

Supplementary Figure S6. Violin plots displaying areas of XRAI heatmap normalized by the whole-image area for (A) Aus, (B) Tak-1/Tak-2, (C) Rit-1/Rit-2. All other settings are the same as in Supplementary Figure S4.

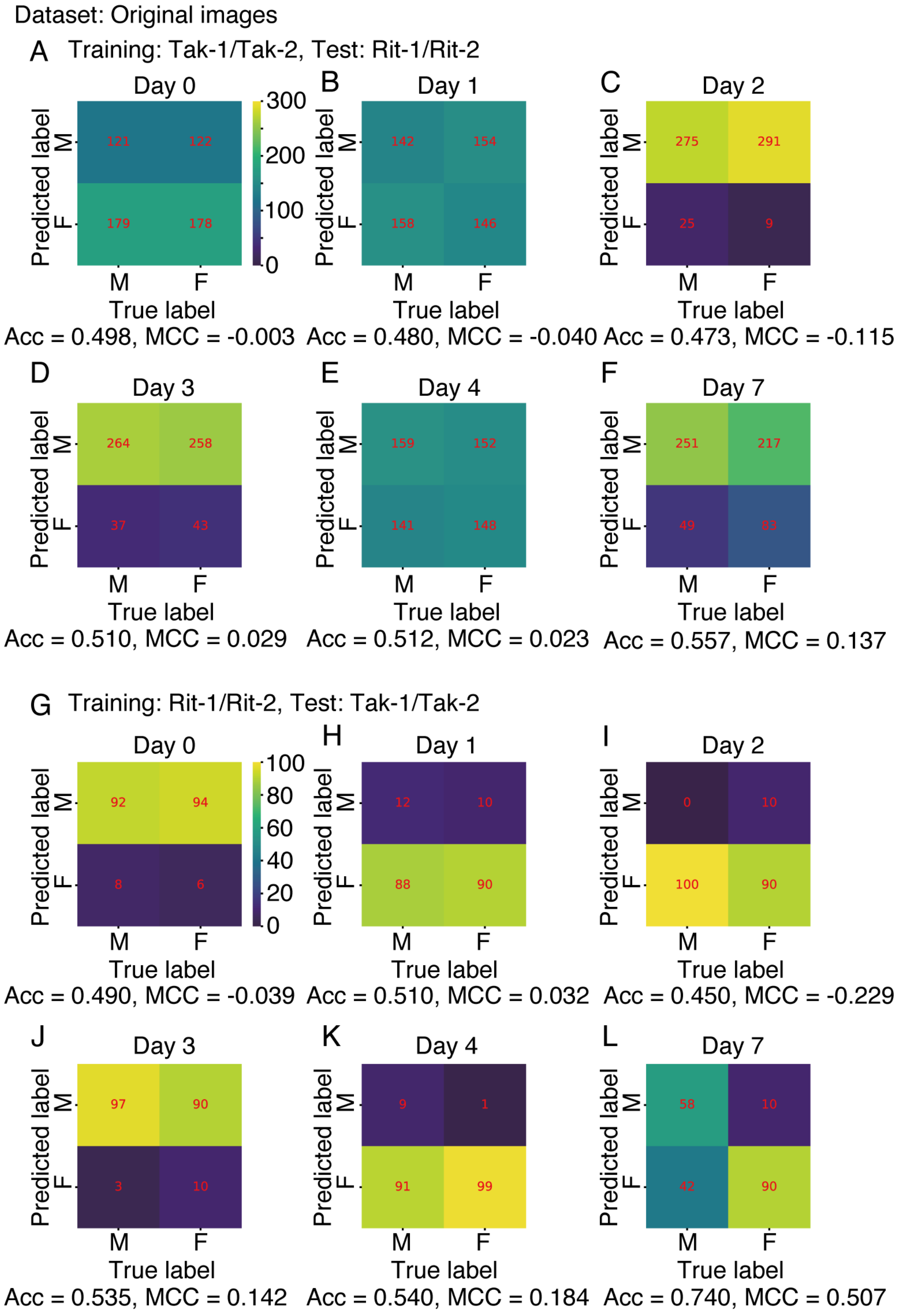

Supplementary Figure S7. Details of transfer prediction in Fig.8. Original non-ablated images were used in training, validation, and testing. (A-F) Performance of Tak-1/Tak-2 classifiers on Rit-1/Rit-2 images. Confusion matrices for true sex labels of Rit-1/Rit-2 plants (columns) and predicted sex labels (rows) for day 0 (A), day 1 (B), day 2 (C), day 3 (D), day 4 (E), and day 7 (F). Numbers in red indicate the numbers of Rit-1/Rit-2 images in the corresponding categories. If the prediction is perfectly accurate for both males and females, the matrix should be diagonal. We also computed the prediction accuracy (acc) and Matthew's correlation constant (MCC) to quantify the model performance. (G-L) Confusion matrices of Rit-1/Rit-2 classifiers on Tak-1/Tak-2 images for day 0 (G), day 1 (H), day 2 (I), day 3 (J), day 4 (K), day 7 (L).

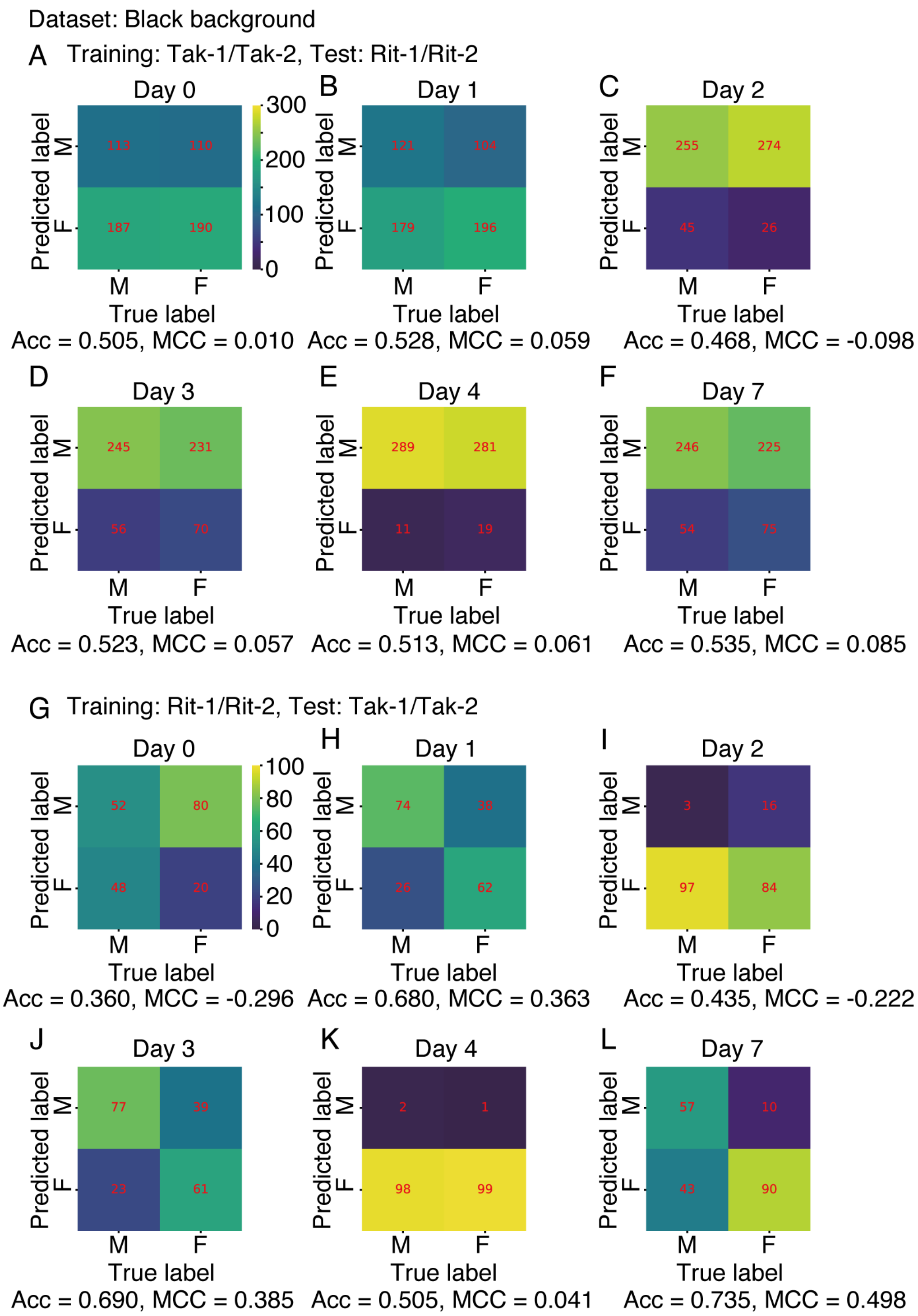

Supplementary Figure S8. Performance of transfer prediction. The background-ablated images, i.e., Ablation (1), were used in training, validation, and testing. All other settings are the same as in Supplementary Figure S7.

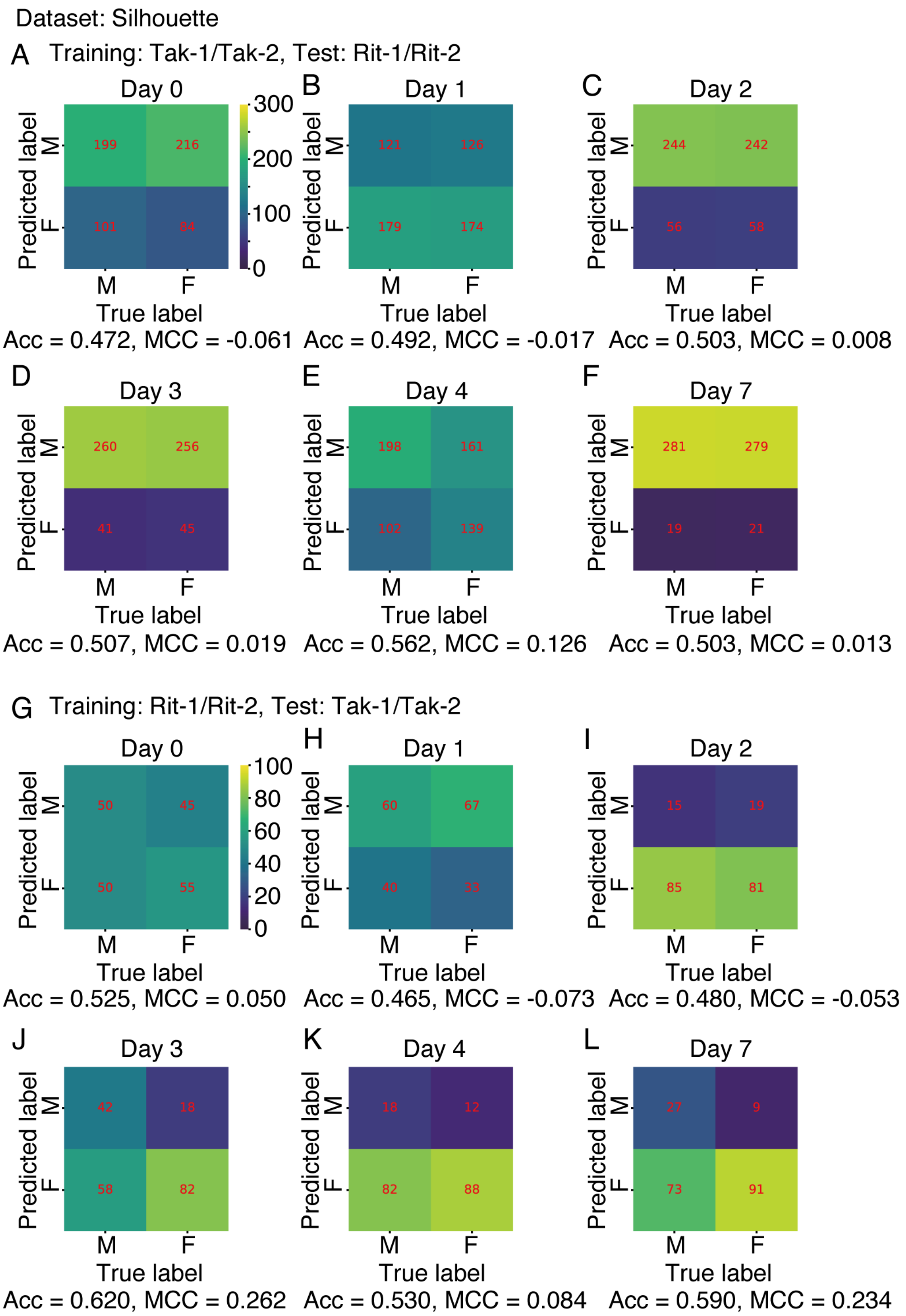

Supplementary Figure S9. Performance of transfer prediction. The silhouette images, i.e., Ablation (2), were used in training, validation, and testing. All other settings are the same as in Supplementary Figure S7.

Supplementary Table S1. Estimated ratios of autosomal regions derived from Tak-1 or Tak-2.

	Tak-1-derived regions		Tak-2-derived regions		Common regions*		Whole length
	Length (Mb)	Ratio (%)	Length (Mb)	Ratio (%)	Length (Mb)	Ratio (%)	(Mb)
Chr 1	11.5	37.6	11.7	38.2	7.4	24.2	30.6
Chr 2	0.4	1.3	8.5	28.6	20.8	70.0	29.7
Chr 3	0.0	0.0	20.1	73.9	7.1	26.1	27.2
Chr 4	8.7	32.2	4.6	17.0	13.7	50.7	27.0
Chr 5	0.1	0.4	3.5	13.1	23.2	86.6	26.8
Chr 6	0.3	1.3	0.1	0.4	23.5	98.3	23.9
Chr 7	4.6	20.9	3.8	17.3	13.6	61.8	22.0
Chr 8	0.1	0.5	11.7	54.7	9.6	44.9	21.4
Total	25.7	12.3	64.0	30.7	118.9	57.0	208.6

The lengths were estimated from the number of 100-kb windows in each category.

Asterisk indicates regions shared between Tak-1 and Tak-2.

Supplementary Table S2. Number of images.

	Aus		Tak		Rit	
day	Male	Female	Tak-1	Tak-2	Rit-1	Rit-2
0	100	100	100	100	300	300
1	100	100	100	100	300	300
2	100	100	100	100	300	300
3	100	100	100	100	301	301
4	100	100	100	100	300	300
7	100	99	100	100	300	300

Supplementary Table S3. Effect size between the sexes, calculated as unbiased Cohen's d.

	unbiased Cohen's d		
day	Aus	Tak-1/Tak-2	Rit-1/Rit-2
0	0.4156	1.3116	0.2208
1	0.2109	1.3929	0.1759
2	0.3093	1.7103	0.0692
3	0.0209	1.5961	0.0231
4	0.3348	1.4969	0.1197
7	1.6032	2.0090	0.1205

Supplementary Table S4. Versions of the libraries used in this study.

Python: 3.6 (in anaconda)<br>PyTorch: 1.7.1<br>Torchvision: 0.8.2
Ubuntu: 16.04<br>Machine: DeepLearning BOX II, 64 GB RAM, GPU=4, GeForce RTX 2080 Ti<br>CUDA driver: 430.34<br>CUDA: 10.1
numpy: 1.18.5<br>scikit-image: 0.16.2<br>scikit-learn: 0.23.1
grad-cam 1.4.8<br>saliency 0.2.0

Supplementary Table S5. Pseudo code for the data transformation.

```
Input: A dataset of original images
Output: A dataset of transformed images
phase: {training, validation, test}

for image in dataset
        if training
                transform= [centercrop(size=1200),
                            horisonalFlip(probability=0.5),
                            verticalFlip(probability=0.5),
                            rotation(min=0 degrees, max=360 degrees),
                            resize(size=(300, 300)),
                            normalize(mean=[0.485, 0.456, 0.406], std=[0.229, 0.224, 0.225])]
                image.transform

        if validation, test
                transform= [centercrop(size=1200),
                            resize(size=(300, 300)),
                            normalize(mean=[0.485, 0.456, 0.406], std=[0.229, 0.224, 0.225])]
                image.transform
```
